# Health status and health service utilization in remote and mountainous areas in Vietnam

**DOI:** 10.1186/s12955-016-0485-8

**Published:** 2016-06-07

**Authors:** Bach Xuan Tran, Long Hoang Nguyen, Vuong Minh Nong, Cuong Tat Nguyen

**Affiliations:** Institute for Preventive Medicine and Public Health, Hanoi Medical University, Hanoi, Vietnam; Johns Hopkins Bloomberg School of Public Health, Baltimore, MD USA; School of Medicine and Pharmacy, Vietnam National University, Hanoi, Vietnam; Institute for Global Health Innovations, Duy Tan University, Da Nang, Vietnam

**Keywords:** Vietnam, Self-rated health, Quality of life, Health service, Utilization, Accessibility, Mountainous, Remote

## Abstract

**Background:**

Self-rated health status and healthcare services utilization are important indicators to evaluate the performance of health system. In disadvantaged areas, however, little is known about the access and outcomes of health care services. This study aimed to assess health-related quality of life (HRQOL), health status and healthcare access and utilization of residents in mountainous and remote areas in Vietnam.

**Methods:**

A cross-sectional study was conducted in a convenient sample of residents in two provinces of Vietnam. Information about socio-economic, health status, HRQOL, healthcare seeking and services utilization were interviewed. EuroQol – 5 Dimensions – 5 Levels (EQ-5D-5 L) was used to measure HRQOL.

**Results:**

Of 200 respondents, mean age was 44.9 (SD = 13.9), 38.0 % were male. One third reported having any problem in Mobility, Usual activities, Pain or Discomfort, Anxiety or Depression. Women tended to suffer more problems in Pain/Discomfort and Anxiety/Depression and lower overall HRQOL than men. Over 90 % of respondents reported at least one health problem. Flu, cold and headache were the most commonly reported symptoms (41.5 %). Most of people preferred community health center when they had illness (96.0 %). Only 18.5 % people used traditional healers with the average of 5.8 times per year. Ethnicity, households’ expenditure, illness and morbidity status, difficulty in accessing health care services were related to HRQOL.; Meanwhile, socioeconomic status, health problems, quality of services, and distances were associated with access to healthcare and traditional medicine services.

**Conclusions:**

Residents in difficult-to-reach areas had high prevalence of health problems and experienced social and structural barriers of healthcare services access. It is necessary to improve the availability and quality of healthcare and traditional medicine services to improve the health status of disadvantaged people.

## Background

Self-reported health status and healthcare services utilization of population are indispensable indicators to assess the performance of health system in the context of limited health administration data [1, 2], particularly in developing countries [2, 3]. They contribute evidences not only to estimate the future demand of healthcare [4], but also to evaluate the health disparities among different groups of people, especially vulnerable subjects such as inhabitants in mountainous and remote areas [1, 3]. This information will help to identify priorities and corresponding solutions to protect and promote health status of the population [4].

Self-rated health status is an important outcomes in primary health care [5], a tool for screening diseases [6] and a reliable predictor of mortality [7]. General health status, measured using a self-rated scale, has been widely used in both clinical trials and population health surveys. However, to better understand the health needs of population, it is essential to incorporate dimensions of health-related quality of life (HRQOL), illness and symptoms [2] as well as health care seeking behaviours and services utilization [8]. Several studies have measured HRQOL of the general population [9] and specific groups in Vietnam, including patients with HIV/AIDS [10–13], chronic conditions [14–16], drug users[11, 12, 17–19], and the elderly [20, 21]. These studies have provided a reference group for future assessment of HRQOL and revealed a high proportion of psychological health problems amongst many patients groups. In addition, people living in the rural and remote areas often perceive poorer HRQOL than those in more advantaged regions [9, 15, 20–24].

The use of health services is associated with not only the availability and quality of services but also preferences of clients which are shaped by their experience, beliefs, health status and socio-economic characteristics [1–4, 25–27]. It has been well documented that health care seeking behaviour is different across regions and socioeconomic status. In developed countries, previous studies reported high prevalence of outpatient clinic visits (e.g., 84 % in Singapore [4], 64.1 % in Taiwan [28]). Meanwhile, most people in developing countries preferred self-medication to treat their disease (e.g., 57–69 % in Vietnam [2, 3], 51.2 % in rural China [27] or 48 % in Thailand’s border [29]). In disadvantaged areas of Vietnam, a study conducted by Toan et al. (2003) showed that only 30 % people used public health services, while this proportion in Thailand’s border was 52 % [29]. Factors associated with public health care utilization in those areas included ethnicity, health status and distance [3, 29].

To promote population’s health and quality of life, Vietnam government emphasizes the role of primary health care, with an emphasis on disease prevention, to achieve “Health for All” as recommended by the World Health Organization. At the grassroots level, community health centres (CHCs) have a responsibility to provide primary health care services, which combine both modern and traditional medicine (TM). TM has a long history and plays an important role in healthcare system of Vietnam [30]. Many people believe that TM is safe and efficacious to use and more accessible than modern medicine [30, 31], especially in difficult-to-reach terrains.

In spite of a large literature about self-rated health and healthcare services utilization in various population, there has been little attention given to communities in mountainous and remote areas [3, 27]. The purpose of this study was to explore HRQOL, health status, healthcare accessibility of remote and mountainous residents in both modern and traditional medicine. The results will help the government develop people-oriented policy for vulnerable population and changing the way to provide health services in difficult-to-reach areas.

## Methods

### Study design and participant recruitment

A cross-sectional study was conducted in two provinces in the north of Vietnam, including Hoa Binh and Quang Ninh. Two communes in resource-limited settings of each province were purposively selected for the survey. In Hoa Binh, Lung Van commune (448 households, 31.5 % poor) and Ngoc My commune (1,341 households, 41 % poor) were selected. In Quang Ninh, Dai Xuyen commune (462 households, 43 % poor) and Van Yen commune (310 households, 31.8 % poor) were selected. These communes are all in mountainous or remote areas and have a distance of 10 to 40 km away from a district health centre. We randomly selected 5 villages in each commune from which we conveniently select 10 households, making a total of 50 households per commune. Well-trained interviewers who were master students at Hanoi Medical University, with support by village health workers, visited households and invited family head or any other people at home to participate in the survey.

### Measures and Instrument

We conducted face-to-face interviews using a structured questionnaire to collect information about patient’s socioeconomic, health status and HRQOL, health care seeking behaviour and health services utilization. The socioeconomic characteristics included age, gender, marital status, education level, employment, and income. Health status and illness, and health services of respondents and other family members use were self-reported, and respondents were asked to show any patient record they had to confirm their illness. In addition, we incorporated other measures of outcomes, including the EuroQOL – 5 Dimensions – 5 Levels (EQ-5D-5 L), a health-related quality of life measure, satisfaction with service quality, and self-evaluated knowledge and competency of using traditional medicine. These patient-reported outcomes includes a set of self-rating questions with higher score indicating more preferable outcomes.

The EQ-5D-5 L includes five dimensions, namely Mobility, Self-care, Usual activities, Pain/Discomfort and Anxiety/Depression, which provided a simple descriptive profile and a single index value for health status [32]. The instrument consists of 2 parts: the EQ-5D-5 L descriptive system and the EQ Visual Analogue Scale (EQ-VAS). The former has five levels of response: no problems, slight problems, moderate problems, severe problems, and extreme problems; and the latter assesses the respondent’s self-rated health on a 20-cm vertical ruler with the endpoint ranging from 0 to 100 points, labelled ‘the worst health you can imagine’ and ‘the best health you can imagine’, respectively [32]. The Vietnamese version of EQ-5D-5 L was translated, culturally adapted and evaluated for its psychometric properties previously [9, 33]. A total of 3,125 health states, which was converted to a single index, were defined by the instrument. In order to calculate the single index, the interim scoring for EQ-5D-5 L from the cross-walk value set of Thailand was used due to the unavailability of Vietnamese population’s preference [32, 33].

### Statistical analysis

Descriptive statistical analysis was used to present the socio-demographics, HRQOL as well as health status (including prevalence of illness amongst respondents) and health seeking behaviours of respondents. Student *t*-test and Chi-squared test were used to compare the difference of those characteristics by gender. The significance level was set at *p* < 0.05.

Multivariate linear regression and logistic regression were performed to determine the factors related to HRQOL (both index and VAS) and difficulty to access health care and TM services. Backward stepwise selection strategy was used to select the models, with variables having p-values of log-likelihood ratio test < 0.1 included and those having p-values > 0.2 excluded [34].

### Ethics, consent and permissions

Written informed consent was obtained from all participants after clearly introducing the survey. Respondents could refuse to participate or withdraw from the interview at any time, and this would not affect their continuation of services. Confidentiality was provided by using coded patient information. Both paper questionnaires and electronic data sets were securely stored.

### Consent to publish

All authors read the manuscript and have consented to publish it.

## Results

### Demographics and health status of respondents

Of 200 respondents, mean age was 44.9 (SD = 13.9), 38.0 % were male, 26.5 % completed high school. Almost all respondents were farmers or self-employed. More than 90 % of households had an annual household income less than US$ 3,000. Two thirds of households reported spending more than 5 % of their total income on health care in the past year (Table 1).

**Table 1:** Socio-demographic characteristics of respondents

	Male		Female		Total		*p*
	Mean	SD	Mean	SD	Mean	SD	
Age	44.67	12.51	45.03	14.71	44.90	13.88	0.43
Age groups (1–5)	N	%	N	%	N	%	
20–30	11	14.47	25	20.16	36	18	0.49
31–40	17	22.37	22	17.74	39	19.5	
41–50	25	32.89	38	30.65	63	31.5	
41–60	16	21.05	20	16.13	36	18	
>60	7	9.21	19	15.32	26	13	
Ethnic	N	%	N	%	N	%	
Kinh	24	31.58	48	38.71	72	36	0.31
Others	52	66.42	76	61.29	128	64	
Education	N	%	N	%	N	%	
Primary school	32	41.51	42	33.87	74	37	0.03
Secondary school	27	35.53	46	37.1	73	36.6	
Above secondary school	11	14.47	9	7.26	20	10	
Others	6	7.89	27	21.77	33	16.5	
Number of family members	N	%	N	%	N	%	
1 to 2	7	9.21	23	18.55	30	15	0.18
3 to 4	37	48.68	51	41.13	88	44	
Above 5	32	42.11	50	40.32	82	41	
Annual income (Vietnamese dong/USD)	N	%	N	%	N	%	
<20 million/1000	29	38.16	63	50.81	92	46	0.08
20–60 million/1000–3000	40	52.63	57	45.97	97	48.5	
>60 millions/3000	7	9.21	4	3.23	11	5.5	
Health expenditure (% of total income)							
<5 %	31	40.79	43	34.68	74	37	0.08
5–10 %	16	21.05	22	17.74	38	19	
10–20 %	20	26.32	24	19.35	44	22	
20–30 %	6	7.89	16	12.9	22	11	
>30 %	3	3.95	19	15.32	22	11	

Self-reported HRQoL of respondents is presented in Table 2. There were 30–40 % respondents reported having any problem in the following dimensions: Mobility, Usual activities, Pain or Discomfort, and Anxiety or Depression. Women (66.1 %) reported a higher proportion of having anxiety or depression than men (23.3 %) (*p* = 0.08). The overall EQ-5D score of respondents was 0.80 (SD = 0.20), and it was higher in men (0.82) than in women (0.78) (*p* = 0.07).

**Table 2:** Health-related quality of life of respondents

	Male		Female		Total		*p*
	N	%	N	%	N	%	
EQ5D items							
Mobility							
Have problems	20	26.32	41	33.06	61	30.5	0.31
No problems	56	73.68	83	66.94	139	69.5	
Self-care							
Have problems	4	5.26	6	4.84	10	5	0.89
No problems	72	94.74	118	95.16	190	95	
Usual activities							
Have problems	24	68.42	42	66.13	66	33	0.74
No problems	52	31.58	82	33.87	134	67	
Pain/discomfort							
Have problems	26	65.79	53	57.26	79	39.5	0.23
No problems	50	34.21	71	42.74	121	60.5	
Anxiety/depression							
Have problems	17	23.37	42	66.13	59	29.5	0.08
No problems	59	77.63	82	33.87	141	70.5	
	Mean	SD	Mean	SD	Mean	SD	
EQ5D index score	0.82	0.20	0.78	0.21	0.80	0.20	0.07
VAS score	74.47	14.57	78.74	67.66	77.12	53.98	0.29

As shown in Table 3, two thirds of households reported that all family members had an illness more than 5 times per year. However, 14.5 % did not seek health care services, and 59.0 % of households used health care services for less than 3 times per year. Of 200 respondents, 91.5 % reported having at least one health problem, and 60.0 % experience more than one health problem. A large proportion of respondents experienced flu, cold or headache symptoms in the past 6 months (41.5 %), followed by musculoskeletal diseases (37 %), and gastrointestinal disease (27 %).

**Table 3:** Health problems of respondents and all family members

	Male		Female		Total		*p*
	N	%	N	%	N	%	
Health status of respondents (last 6 mo.)							
No disease	10	13.16	7	5.65	17	8.5	0.11
One disease	22	28.94	41	33.06	63	31.5	
Two diseases	31	40.79	63	50.81	94	47.0	
More than two diseases	13	17.11	13	10.48	26	13.0	
Type of health problems							
Flu, cold, fever or headache symptoms	29	38.16	54	43.55	83	41.5	0.45
Cardiovascular disease	10	13.16	12	9.68	22	11	0.44
Respiratory disease	17	22.37	18	14.52	35	17.5	0.16
Gastrointestinal disease	20	26.32	34	27.42	54	27	0.86
Musculoskeletal disease	26	34.21	48	38.71	74	37	0.52
Others	22	28.95	42	33.87	64	32	0.47
Frequency of having illness and health problems by all family members							
<5 times	30	39.47	42	33.87	72	36	0.65
5–9 times	15	19.74	34	27.42	49	24.5	
10–15 times	24	31.58	36	29.03	60	30	
>15 times	7	9.21	12	9.68	19	9.5	
Frequency of seeking health care services by all family members							
Not need	10	13.16	19	15.32	29	14.5	0.45
1–3 times	41	53.95	77	62.1	118	59	
4–5 times	17	10.53	18	14.52	35	17.5	
>5 times	8	9.21	10	8.06	18	9	

### Health services access and utilization

Table 4 describes health care seeking behaviours and accessibility to health care services among respondents. Community health center was the level that respondents visited most frequently once they have a health problem (96.0 %), followed by district hospitals (42.0 %). There were 18.5 % of respondents seeking traditional healers for their health care, and on average, respondents used traditional medicine 5.84 times per year. However, more than 20 % of respondents reported that they had self medication without consultation to health workers. Inaccessibility to general health care services was still prevalent among this group, accounting for 29.5 %. Meanwhile, inaccessibility to traditional medicine services was only 11 %.

**Table 4:** Health seeking behaviours of respondents

	Male		Female		Total		*p*
	N	%	N	%	N	%	
Health services utilizations							
Community health center	71	93.42	121	97.58	192	96	0.14
District hospital	26	34.21	58	46.77	84	42	0.08
Province hospital or above level	19	25.00	18	14.52	37	18.5	0.61
Private clinic	5	6.58	8	6.45	13	6.5	0.97
Traditional healers	11	14.47	26	20.97	37	18.5	0.45
Self-treatment	19	25.00	24	19.35	43	21.5	0.35
Accessibility to health service							
Difficult to access health service	21	27.63	38	30.65	59	29.5	0.65
Difficult to access traditional medicine service	9	11.84	13	10.48	22	11	0.77
	Mean	SD	Mean	SD	Mean	SD	
Traditional medicine package use							
Frequency of use (times/yr.)	6.29	4.40	5.56	3.25	5.84	3.73	0.09
Frequency of refiling the traditional medicine	3.47	2.74	3.02	1.86	3.20	2.24	0.08

Table 5 presents factors associated with HRQoL of respondents which was measured using EQ-5D-5 L and EQ-VAS. We found that significantly higher EQ-5D-5 L index was observed among Muong ethnic people (compared to Kinh people) and among those with higher annual income. In addition, illness and morbidity is a significant predictor of lower health-related quality of life. Having one or multiple health problem(s) resulted in a decrement of 0.065 to 0.102 score in EQ-5D-5 L index. Regarding the VAS score, we found that difficulty in accessing health care services and health care spending were two significant predictors of poorer health-related quality of life among respondents.

**Table 5:** Factors associated with health-related quality of life of respondents

	EQ–5D-5 L Index				VAS score			
	Coef	*p* value	95 % CI		Coef	*p* value	95 % CI	
Age groups (1–5)	−0.061	<0.01	−0.081	−0.041				
Sex: female vs. male					11.41	0.16	−4.46	27.28
Ethnics								
Kinh (ref)								
Muong	0.11	<0.01	0.049	0.17				
Others	0.059	0.12	−0.015	0.133				
Annual income per head								
Lowest (ref)								
Low					15.53	0.13	−4.41	35.46
Middle								
High	0.091	0.01	0.02	0.162				
Highest	0.102	0.01	0.027	0.177				
% Spending on health care								
Lowest (ref)								
Low	0.065	0.06	−0.002	0.131				
Moderate					−26.86	0.01	−6.84	−46.88
Difficulty in accessing health services								
Yes vs. No					−16.54	0.05	−32.84	−0.23
Distance to health station								
<1 km (ref)								
1–2 km	−0.052	0.06	−0.106	0.002	−12.4	0.13	−28.47	3.66
Health problems								
None (ref)								
Single	−0.065	0.19	−0.163	0.032				
Multiple	−0.102	0.03	−0.196	−0.009				
Const	0.972	<0.01	0.863	1.082	72.36	<0.01	58.07	86.65

(Full model included age, sex, religion, educational attainment, annual income per head, health problems, health care spending, and distance to health stations, difficulty in accessing health services, and difficulty in accessing traditional medical care services)

In Table 6, we explored factors associated with reported difficulties in accessing health care and traditional medicine services in logistic regression models. In general, we found that respondents who had better economic status, lived further away from CHC, perceived poorer quality of health services, and unsatisfied with services availability were more likely to report difficulties in health service access. Comparing Kinh with Muong people, we found that the number of health problems and distance were the two major factors associated with health service access among Kinh people, meanwhile in Muong people, lower education and satisfaction with TM predicted having difficulty with health service access. Regarding use of TM services, it was different between Kinh and Muong people. Among Kinh people, poorer perceived quality of commune health service was associated with less difficulty in TM access; however it was “borderline” significant (*p* < 0.1). Among Muong people, difficulty in TM access was positively associated with better economic status (measured using household’s expenditure), dissatisfaction with service availability, perceived TM as less effective and having problems in daily activities. Meanwhile, it was negatively associated with having problems in Anxiety or Depression. Barriers to health services access included long distance to health care facilities (19 %), poor health services quality (3 %), and unaffordability (14 %); meanwhile limited access to traditional medicine was primarily due to the unavailability of demanding services and drugs (6 %). Hence, if it is deemed desirable to increase the use of CHC services, it will be necessary to bring the CHC nearer to the households.

**Table 6:** Factors associated with having difficulty in accessing health services and traditional medical care services

Factors	Difficulty in general health services access						Difficulty in traditional medicine and services access					
	All		Kinh		Muong		All		Kinh		Muong	
	OR	95 % CI	OR	95 % CI	OR	95 % CI	OR	95 % CI	OR	95 % CI	OR	95 % CI
Age (years)			1.1*	(1.0; 1.1)					1.1	(1.0; 1.2)		
Male vs. Female			0.3	(0.1; 1.4)	3.1*	(1.0; 10.2)						
Ethnic: Kinh vs Muong							4.3**	(1.1; 16.3)				
Education (Elementary - ref)												
Secondary	0.5	(0.2; 1.2)										
College and above	2.1	(0.7; 6.5)	0.2	(0.0; 1.7)	10.6***	(2.7; 41.5)	5.0**	(1.5; 17.4)				
Expenditure (Lowest - ref)												
High	0.5	(0.2; 1.4)									3.1*	(0.8; 11.7)
Highest	5.8***	(1.6; 20.7)									8.4*	(1.0; 71.6)
Household’s income (Lowest - ref)												
Low	2.1	(0.8; 5.7)	4.3	(0.5; 36.5)			4.5**	(1.2; 16.4)				
Middle			0.1*	(0.0; 1.2)								
Highest	0.4	(0.1; 1.5)										
Having problems in (Yes vs. No)												
Daily activities											5.5**	(1.1; 26.6)
Pain or Discomfort							5.4***	(1.7; 17.5)			3.9*	(1.0; 15.6)
Anxiety or Depression							0.1***	(0.0; 0.6)			0.1**	(0.0; 0.8)
# health problems (None - ref)												
1 health issue	6.0*	(1.0; 37.0)	17.6**	(1.1; 294.0)							2.4	(0.7; 8.9)
2 health issues	3.9	(0.7; 22.3)	18.5**	(1.2; 285.1)								
Distance (<1 km – ref)												
>2 km	3.6***	(1.5; 8.3)	12.8***	(2.1; 79.2)	2.6	(0.8; 8.3)						
Perceived quality of commune health services												
Very good - ref												
Good	3.0	(0.8; 12.1)							0.1*	(0.0; 1.0)		
Moderate	6.0**	(1.0; 34.4)										
Perceived of TM service quality (Very good – ref)												
Good							6.1**	(1.0; 36.7)				
Perceived of effectiveness of TM (Very good - ref)												
Good							0.4*	(0.1; 1.1)			1.2**	(1.0; 1.5)
Moderate	0.3*	(0.1; 1.1)	0.2	(0.0; 1.8)								
Satisfaction with (Yes vs. No)												
Availability of health services	0.2***	(0.1; 0.7)			0.3	(0.1; 1.5)					0.1**	(0.0; 0.7)
Commune general health care services											3.4	(0.7; 17.3)
Commune traditional health care services					0.1**	(0.0; 0.6)						
Health information and guidance							6.9**	(1.1; 42.5)				
Convenience of service use					3.3*	(0.8; 13.0)						
Convenience of integrating western and traditional health services	0.5	(0.2; 1.3)										
Inter-professional collaborations					6.4	(0.7; 61.9)						
Capacity of health workers					0.2*	(0.0; 1.4)						
Responsiveness of health workers							0.1**	(0.0; 0.8)				

*** *p* < 0.01, ** *p* < 0.05, * *p* < 0.1; 95 % CI in parentheses

## Discussion

This study indicated high proportions of health problems across five dimensions of health-related quality of life among people living in remote and mountainous areas in two provinces of Vietnam. In addition, we found that accessibility and utilization of health care services were not sufficient and associated with various social and structural factors. This included household’s economic status, severity of health problems, health care costs, distance to and quality of health and traditional medical services.

The findings demonstrated that women perceived lower HRQOL and more anxiety/depression problems than men. It confirmed findings from previous studies in Vietnam and worldwide [10, 15, 35, 36]. In Vietnamese tradition, women take primary responsibility for taking care of their children and family. Besides, in mountainous or remote areas with poor infrastructure and know-hows for economic development, people have to work hard to feed their family. Those burdens may contribute to a higher proportion of having problems in mental health and lower HRQOL in women than men. In our sample, we found better HRQOL among those who had higher income, meanwhile, health care expenditure, difficulties in accessing health services, and comorbidity were significantly affecting HRQOL of respondents. These results were also found in a study of Topal et al. [37], which was conducted on Turkish immigrants - a vulnerable population, in London, United Kingdom.

When investigating health-seeking behaviours, the findings suggested that CHC was the most preferable health facilities of respondents. CHC is the closest local station that provides primary care and prevention programs [38]. The Vietnamese principles of health system operating include the integration of modern and traditional medicine at grassroots level as well as health promotion programs [39]. However, the underuse of health services and high frequency of self-medication observed in this study could be related to long distance, quality and availability of demanding services [40]. Additionally, the results of multivariate analysis revealed that distance to health facilities was a remarkable determinant of health service utilization, which is also comparable to observations in previous studies conducted in Vietnam [3, 41, 42] and other countries such as Nepal [43], Ghana [44], China [27] and United States [45]. Poor quality of roads, lack of transportations, and travel costs are found to be significant barriers to health care access.

The results also indicated the role of quality care perception as a predictor of healthcare utilization. A study conducted by Nguyen et al. [46] examining CHC utilization in remote and poor Vietnam communes demonstrated that after enhancing service’s quality, the utilization rates of population were improved significantly. Duong et al. [47] had similar results when investigating factors associated with delivery services among women in rural Vietnam. They also underlined the major impact of provider-client relationships on the quality of services. In Nigeria, Obiechina and Ekenedo found that satisfaction with services was a factor affecting health service utilization in university [48].

It is noteworthy that although one third of respondents reported difficulties in accessing health services, about ninety percent of their sample did not have significant obstacles to approach traditional medications. TM plays an important role in of Vietnamese culture and health system [30]. In accordance to our observation, these residents tended to use TM or go see traditional healers, which are available in their villages.

Differences in services access were found amongst different ethnic groups, specifically, Muong people accessed health care service easier than the ethnic majority (such as Kinh people), but more difficult in access to TM. This finding was different from previous studies. Toan et al. suggested that ethnic minority‘s attendance to public health services was lower than the ethnic majority due to their limited language and transportations [3]. However, in our study setting, for example Hoa Binh, majority of health care workers at a CHS were ethnic people or fluent in local languages. This may reflect the progress in government’s efforts to improve quality and coverage of health care services for the poor and disadvantaged areas over the past decade. Nonetheless, a recent study by Malqvist et al. [49] indicated that the ethnicity-based discrimination were still existed in Vietnam, resulting in the lower utilization of the minority ethnic compared to their counterparts.

This is the initial study to emphasize the health status and disparities in health care and TM access among vulnerable population namely Vietnamese mountainous or remote residents, which has been lack of evidence in literature. The data partly contributes to some implications. First, support of communities, encouragement of family and promotion of women’s empowerment are potential solutions to improve quality of life of women [50]. Second, enhancing quality of care, such as enhancing personnel, equipment and facility, could encourage people using health services in CHC in mountainous or remote areas. Nguyet et al. (2012) suggested that whether in poor or less poor communes, the use of CHC will be increased if services meet the minimal standard [46]. Finally, CHC could develop TM services in parallel with modern medicine and guide the residents to use this available medication appropriately. Future studies should explore potential socioeconomic and structural barriers to the access and use of TM services that are provided at commune health stations for common health problems in the rural and mountainous areas.

The study’s strengths comprise the investigation in vulnerable subjects within large geographical areas. In addition, multivariate analysis was used appropriately to adjust the confounders and find the related factors with health status and health utilization. However, the study has several limitations. First, results were obtained from a cross-sectional study, therefore no causal association was established for health status and health seeking behaviours (include TM access). A longitudinal analysis should be required to explore the causal effects among socio-economic factors, health outcomes and health utilization. Second, the data was only collected in a convenient sample, which may not be representative of mountainous and remote areas in Vietnam. Further research in other mountain areas is needed to provide comprehensive evidence for policy-makers. Third, although patient records were required to confirm if respondents had any illness examination in the past, the results mainly based on self-reported data which may subject to recall bias. Integrating self-reported information and clinical examination or health record diary should be conducted for more reliable assessment. Moreover, it is noteworthy that the sample is slightly skewed towards women (62 %) since more women were at home and thus available to be interviewed. This might affect the representativeness of reported health problems since women might perceive their health status different than men. Finally, some factors such as knowledge, attitude and practice of respondents about disease prevention and TM usage were not comprised in this study. Examining those factors may help to develop further appropriate interventions for this vulnerable population.

## Conclusion

In conclusion, these data showed the high prevalence of health problems and low proportion of people approaching healthcare services in Vietnam mountainous and remote areas. Large population preferring community health centre and accessing easily traditional medication were suggestions to develop TM services in grassroots health level, especially in mountainous and remote areas.

## Abbreviations

CHC, community health centre; EQ-5D-5 L, EuroQol- 5 Dimensions – 5 levels; HRQOL, health-related quality of life; TM, traditional medicine.
